# Annual Reviews 2020: Recent advances in analytical sciences

**DOI:** 10.1002/ansa.202100013

**Published:** 2021-04-09

**Authors:** Sebastiaan Eeltink

**Affiliations:** ^1^ Department of Chemical Engineering Vrije Universiteit Brussel Brussels Belgium

This issue of *Analytical Science Advances* includes review papers written by authorities in the field, covering a wide range of topics in the field of analytical sciences. Authors were asked to provide a critical review discussing the state‐of‐the‐art in their field and highlight the key developments and applications covering the literature published in 2020. These reviews are ideally suited for those who want a rapid overview of the latest advances in a particular field and learn about emerging applications.

Mass spectrometry (MS) is an immensely important analytical technology that still rapidly develops and continues to find new application areas. In their review on the rise of single‐cell proteomics, Ctortecka and Mechtler discuss the current state‐of‐the‐art MS‐based workflows. In particular, aspects of sample preparation when working with minute amounts of samples are discussed, new possibilities in separation science are highlighted, and emerging MS quantification strategies including data postprocessing are discussed. An overview of the current capabilities of high‐resolution MS and its applicability to pharmaceutical analysis is provided by Géhin and Holman. Rankin‐Turner and Heaney discuss new technological advances in ambient ionization MS and provide an overview of key paper published in biomedical sciences, forensics and security, food sciences, the environment, and chemical synthesis. The Hoffmann group summarizes the latest developments in MS imaging, with a focus on the latest approaches for tissue‐based imaging.

Several topics are covered in the field of separation science. De Vos et al. summarize the latest advances in UHPLC and multi‐dimensional LC instrument development and highlight recent key applications. The Franchina group reports on the latest instrumental advances and applications of comprehensive two‐dimensional gas chromatography (GC × GC), including its hyphenation with novel upstream or downstream processes. Major applied fields such as energy, fuel, foodstuff, plant, biological, and environmental are also covered. The analysis of low molecular weight polar and ionic components using ion chromatography (IC) coupled to MS is summarized by Bruggink and Jensen. Further applications using IC hyphenated to inductively coupled plasma mass spectrometric detection are reviewed. Muza et al. described the recent advances in designing and fabricating bioconjugated and self‐assembled polymer structures and their characterization using field flow fractionation. The latter separation technique utilizes fluid flow dynamics and external force fields for effective separations at steady‐state conditions within a solvent filled channel. Finally, Eeltink et al. reviewed recent advances in the field of preparation and application of organic polymer‐based monolithic stationary phases.

Other topics included in this review issue are high‐throughput experimentation (HTE), the analysis of volatile flavor and fragrance compounds, and trends in artificial intelligence, machine learning, and chemometrics applied to chemical data. HTE is a growing and evolving field, and the review by Vervoort et al. considers throughput of chromatographic and spectroscopic techniques, as well as advances in sample manipulation. The analysis of volatile flavor and fragrance compounds is essential when characterizing food and environmental samples, essential oils, and cosmetics. Louw reviewed the techniques that were most frequently used in 2020, which comprises solvent assisted flavor evaporation for extraction, GC coupled ion mobility spectrometry, and electronic senses. Houhou and Bocklitz reviewed artificial intelligence based methods including chemometrics, machine learning, and deep learning for the analysis of chemical and spectroscopic data. In this respect, inverse modeling, preprocessing methods, and data modeling applied to spectra and image data for various measurement techniques are discussed, allowing to translate measurements such that data can be used to its full extent.

Recently, *Analytical Science Advances* launched an "Art in science" initiative, where we showcase artwork linked to a scientific publication.^1^, ^2^ For this issue, I have approached Calissa Seelen from Caligrafix Productions and asked her to create an artist impression covering different topics presented in this review issue. Calissa obtained a Bachelor of Law in The Netherlands and a Master in Sociology in Belgium. She developed a passion for analyzing and researching social inequality and aspects that combines law and sociology. After her studies she worked at the European Union Agency for Fundamental Rights in Vienna (Austria). There she experienced first‐hand that all the amazing, life‐changing, and international research that this prestigious institute conducted did not reach the common people of the EU. After moving back from Vienna to Belgium, Calissa started Caligrafix Productions. Among others, she creates digital illustrations, logos, and layouts for the sole purpose of improving the effective communication between the sender of the message and its receiver. Research findings are an extremely important tool to change the world we live in today. One finding or result can impact many people in their (daily) lives, without them even knowing how that a concept or technology became reality.

**FIGURE 1:**
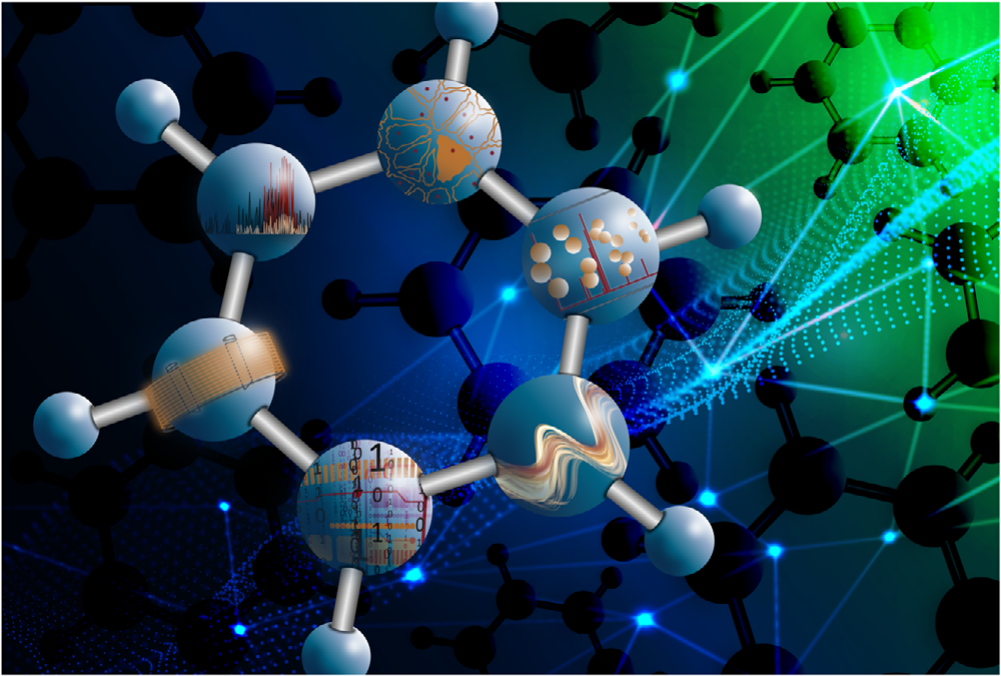
Illustration by Calissa Seelen from Caligrafix Productions; www.caligrafix.be covering different topics presented in this review issue

Figure 1 shows the digital artwork created by Calissa. The reasoning behind this design is explained by the illustrator:

The main idea for me was to create an illustration that contains the essence of the general subject of the journal, as well as the different thematic areas in which the specific papers operate. Through this digital art image, I wanted to show that we as humans are all interconnected through our lives, interests, and research findings. This state of interconnectedness is shown on the background of the image. It shows a networking structure based on the theory of six degrees of separation as first researched by Stanley Milgram in his Small World Experiment,^3^ which was later referred to as the groundwork of this theory. It is a common concept used in the field of social psychology and widely used in other social sciences. It hypothesizes that any person can be connected to any other person through a chain of acquaintances that has no more than five intermediaries on average. The background illustration also represents the need for interdisciplinary research and collaboration between researchers worldwide. The dotted wave in the background is moving toward the lighter area of the image in the upper right corner and represents our efforts to get out of the "dark" or "unknowing" and finally reach a state of understanding, unity, and equality among each other. Lastly, there are molecules in a three‐dimensional area in the background, interfacing the network structure, which again represents interconnectedness between the small things of this world and the people who study them.

The illustration on the foreground represents a molecular structure where every atom highlights a specific paper from this special issue. I will explain the meaning of each atom in a clockwise manner. The first atom situated at the top of the molecule is based on the paper by Ctortecka and Mechtler.^4^ I wanted to create a simplified version of a human cell structure, highlighting the hunt for the proteome from a single cell where modern proteomics approaches are used. It is literally the light in the dark, the target that was hunted for decades and now finally comes within reach. The second atom is linked to MS correlated with reviews by Rankin‐Turner and Heaney,^5^ Géhin and Holman,^6^ and the Hoffmann group.^7^ It represents the essence of time‐of‐flight MS analysis, where molecules are drifting in a vacuum toward the detector. Upon detection, the mass spectrum of the compound of interest is generated as shown in red. The next atom showcases the paper by Louw.^8^ It depicts fragrance molecules that diffuse in air. The shape of the strokes also represents the peaks that show up during analysis in, for example, GC. The tinner red band is the small string of aromatic compounds that the researchers are aiming to discover. The fourth atom is inspired by machine learning and binary codes to demonstrate the following paper: Trends in artificial intelligence, machine learning, and chemometrics applied to chemical data by Houhou and Bocklitz.^9^ The fifth atom is based on the paper by Franchina et al.^10^ It shows the capillary column placed at the center of the molecule as the column is considered the heart of any gas chromatography analysis. Furthermore, it shows that over the course of several decades the core of the technology has apparently not changed a lot (i.e., the columns still look exactly the same) but the technique is so powerful that it definitely deserves to be highlighted. The last atom shows a peak pattern that needs to be resolved based on the paper by De Vos et al.^11^ The three different "overlapping" chromatograms show how information from multiple dimensions of separation are required to provide the required information.

As a whole, the work from these different experts is interconnected and completes the picture of the molecule that makes up the most important part of the illustration. Without these different innovations, fundamental knowledge in chemistry and life sciences could not be obtained and the illustration would lose its value. By symbolizing the unity between the technological advances, I want to stress once more the importance of multidisciplinary alliances, which is the future for scientific discoveries.

It has been a great pleasure to complete this special issue. I would like to thank all contributors for their valuable time to write these excellent reviews. Clearly, great progress is being made in the field analytical sciences, and the techniques and methods developed have a direct impact on society. I am looking forward for a follow‐up issue that will be published in 2022 that may include recent highlights of the current themes and also provide insights in new emerging topics.
